# Time zero determination for FEL pump-probe studies based on ultrafast melting of bismuth

**DOI:** 10.1063/1.4999701

**Published:** 2017-10-26

**Authors:** S. W. Epp, M. Hada, Y. Zhong, Y. Kumagai, K. Motomura, S. Mizote, T. Ono, S. Owada, D. Axford, S. Bakhtiarzadeh, H. Fukuzawa, Y. Hayashi, T. Katayama, A. Marx, H. M. Müller-Werkmeister, R. L. Owen, D. A. Sherrell, K. Tono, K. Ueda, F. Westermeier, R. J. D. Miller

**Affiliations:** 1 Max Planck Institute for the Structure and Dynamics of Matter, 22761 Hamburg, Germany; 2 Graduate School of Natural Science and Technology, Okayama University, Okayama 700-8530, Japan; 3 Institute of Multidisciplinary Research for Advanced Materials, Tohoku University, Sendai 980-8577, Japan; 4 RIKEN SPring-8 Center, 1-1-1 Kouto, Sayo-cho, Sayo-gun, Hyogo 679-5148, Japan; 5 Diamond Light Source, Harwell Science and Innovation Campus, Didcot OX11 0DE, United Kingdom; 6 The Hamburg Centre for Ultrafast Imaging, Luruper Chaussee 149, 22761 Hamburg, Germany; 7 Japan Synchrotron Radiation Research Institute, 1-1-1 Kouto, Sayo-cho, Sayo-gun, Hyogo 679-5198, Japan; 8 Departments of Physics and Chemistry, University of Toronto, Toronto, Ontario M5S 1A7, Canada

## Abstract

A common challenge for pump-probe studies of structural dynamics at X-ray free-electron lasers (XFELs) is the determination of time zero (T_0_)—the time an optical pulse (e.g., an optical laser) arrives coincidently with the probe pulse (e.g., a XFEL pulse) at the sample position. In some cases, T_0_ might be extracted from the structural dynamics of the sample's observed response itself, but generally, an independent robust method is required or would be superior to the inferred determination of T_0_. In this paper, we present how the structural dynamics in ultrafast melting of bismuth can be exploited for a quickly performed, reliable and accurate determination of T_0_ with a precision below 20 fs and an overall experimental accuracy of 50 fs to 150 fs (estimated). Our approach is potentially useful and applicable for fixed-target XFEL experiments, such as serial femtosecond crystallography, utilizing an optical pump pulse in the ultraviolet to near infrared spectral range and a pixelated 2D photon detector for recording crystallographic diffraction patterns in transmission geometry. In comparison to many other suitable approaches, our method is fairly independent of the pumping wavelength (UV–IR) as well as of the X-ray energy and offers a favorable signal contrast. The technique is exploitable not only for the determination of temporal characteristics of the experiment at the interaction point but also for investigating important conditions affecting experimental control such as spatial overlap and beam spot sizes.

## I. INTRODUCTION

The stochastic nature of the radiation process leading to lasing of present X-ray free-electron-lasers (XFELs)^1^ imposes an intrinsic arrival time jitter (ATJ) on the pulses at the experimental interaction point with respect to the nominal time basis of the FEL or any other precisely periodic reference, e.g., another pulsed laser source. Unfortunately, a vast class of experiments performed at FEL facilities indeed rely on a known and experimentally accessible delay between the FEL and an experiment specific laser light source. These experiments are commonly referred to as pump-probe experiments.

Over the past few years, there have been efforts to reduce this jitter experimentally at its origin,^2,3^ tackle it by a mathematical algorithm^4^ to gain better timing resolution, or simply measure the ATJ for every FEL shot and subsequently correct each data point during data analysis. Many different approaches have been carried out to measure single shot based ATJ. Such setups, when employed at FEL related experiments, are commonly referred to as timing tools. The challenge to all these approaches is the requirement to sparingly use the FEL pulses for T_0_ measurements, to exploit most of the XFEL photons for the actual experiment while draining only a small fraction of the totally available FEL power for measuring the ATJ. A frequently suggested and also vastly applied scheme exploits detection of changes of reflectivity—or to a lesser extent transmission—of a solid, which is pumped by the FEL light and probed by an additional infrared laser around 800 nm wavelength (or eventually its second harmonic).^5–16^ Other schemes employ terahertz (THz) radiation on solid as well as gaseous targets^17–19^ in very distinct ways including the use of THz radiation created by the same electron beam which emits X-rays in the undulator section of the FEL. Gaseous targets have been also exploited for ATJ correction by aid of other types of optical radiation.^20–22^ Somewhat different but occasionally successfully employed approaches are based on electro-optical sampling.^23–25^ Finally, very interesting classes of experiments utilize the changes in the Bragg peak intensities of crystalline solids due to bond elongation and contraction as a consequence of a photon-phonon interaction of the pump laser with the crystal lattice.^14,26,27^

Having a timing tool in place providing ATJ uncertainties well below 100 fs is basically a necessity for a successful pump-probe experiment at any FEL facility. However, such timing tools generally deliver only timing information between the pump laser and the FEL at the position of the timing tool. That position is usually not the experimental interaction point, and the (unknown and differing) path lengths to the interaction point typically differ for the optical laser and FEL. Hence, the information the timing tool provides is only relative and thus suitable for accurately identifying shot-to-shot variations in the pump-probe delay at the interaction point but not the delay itself. A remaining unknown to be determined for every experiment is the temporal overlap, or pump-probe time, at the most important position—the interaction point of the experiment. A task, which can synonymously be described as finding the time T = 0 (T_0_). Once T_0_ at the interaction point has been determined, the timing tool is in principle calibrated, in the sense that the continuously monitored single shot pump-probe delays at the timing tool position can be easily converted to absolute pump-probe times at the interaction point of the experiment.

There are a few schemes to realize a timing tool, some of which are made readily available by the FEL facilities.^8,16^ The obviously equally important task to find T_0_ has not been targeted in a comparably general approach. One of the reasons is certainly that the necessary access to the interaction point is much more experiment specific compared to the rather general case of a timing tool setup at some dedicated spot at the FEL beamline. The most frequently employed timing tool concepts exploit ultrafast optical changes of GaAs or silicon nitride samples. Typically, the roles of FEL and optical laser are reversed, and the samples are pumped by the FEL and probed by the optical laser, of which the reflectivity or transmissivity is measured as a function of pump-probe-delay. An optical wavelength of around 800 nm seems to be a sweet spot for these experiments. One reason is certainly that femtosecond Ti:Sapphire lasers, a frequent choice in typical pump-probe setups with and without a FEL contribution, operate with their fundamental wavelength around 800 nm. A second reason is constraints from the band gap of the employed timing sample. GaAs, for example, has a bandgap of 1.43 eV with a strongly increasing absorption for photons above the band gap and hence constrains its use to certain wavelengths.

Most of the concepts exploited in timing tool setups could probably be used for timing at the interaction point and hence to determine T_0_. The T_0_-timing needs to be checked repeatedly during an experimental campaign, since it is one of the most crucial parameters for the interpretation of dynamical data. Therefore, a setup to measure T_0_ should allow for a quickly performed integration into the actual experimental setup at the interaction point. It is of importance to perform the two measurements, namely, the T_0_ determination and the actual experiment using generally a very different sample, at the same point in space. Without countermeasures, movements can introduce considerable timing shifts between the two beams, a problem more pronounced in non-collinear geometries between pump laser and FEL axes (which is typical). It is also advantageous, if the T_0_-setup operates very close to the parameter space of the main experiment to be performed, e.g., in respect of optical laser and FEL wavelength, beam dimensions, and intensities. In existing and future experiments, the optical pump wavelength to trigger dynamics within a sample can span a wide range of wavelengths from the far-IR to the ultra-violet. From this perspective, a rather general approach to determine T_0_ at the sample position is desirable—applicable to the largest possible sub-set of potential optical wavelengths.

Most important, there is the possibility to use the dynamics of the target under investigation to determine T_0_. The faster the dynamics of that particular sample, the better are the prospects to determine T_0_ sufficiently well by investigating the sample itself at small time delays. However, in cases where the response of the target is delayed, or for some reasons very weak, e.g., because the tolerable pumping fluence is restricted and needs to be kept low, such intrinsic approaches are hampered or not even applicable.

**FIG. 1.**
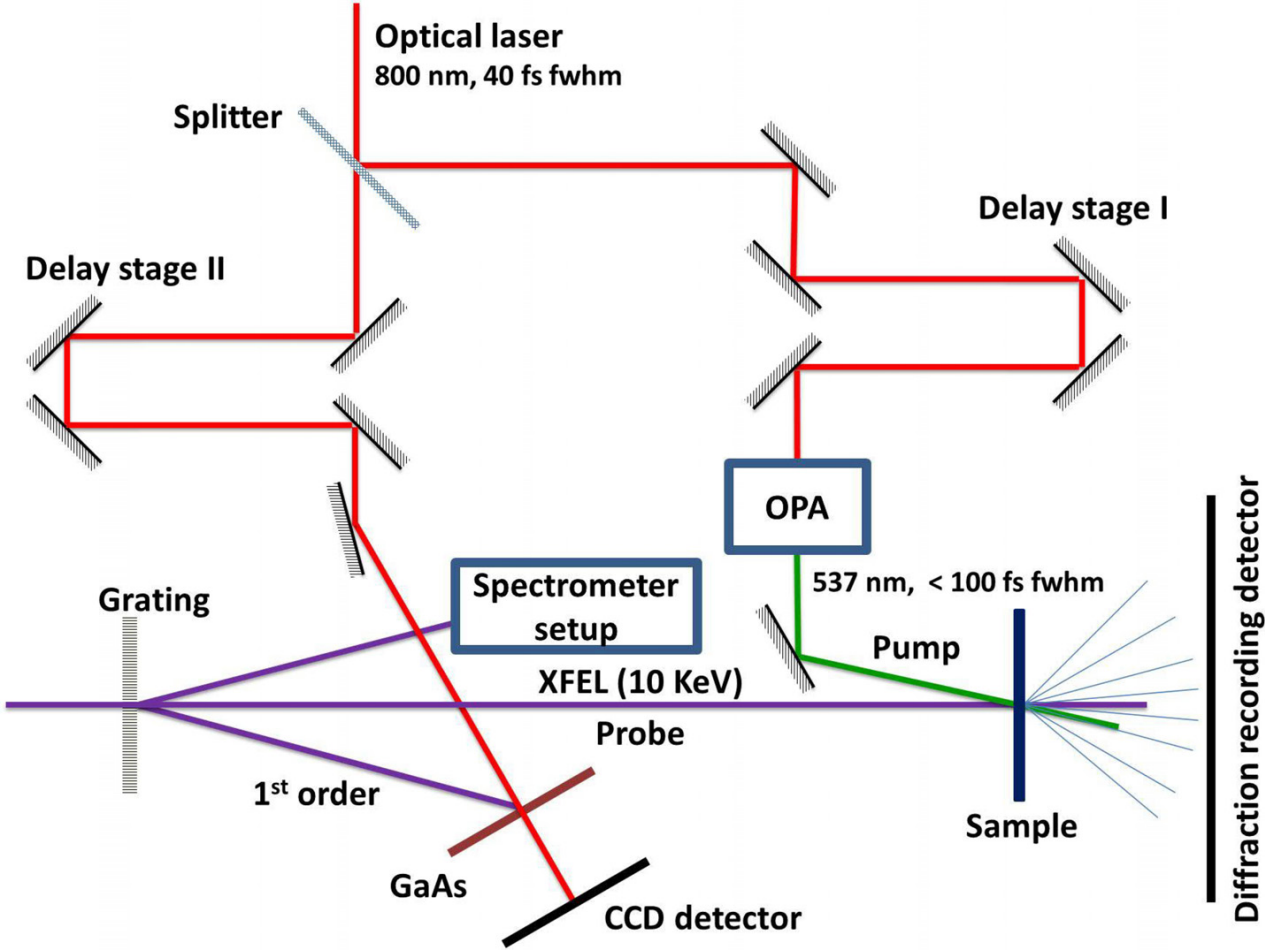
Schematic beam paths of the experiment. The optical laser branch is split into two parts. Both parts employ a delay stage to set the relative timing to the FEL independently. The left optical branch serves the facility timing tool.^8^ The right branch is used as a pump pulse for the sample. After passing an X-ray grating upstream of the sample interaction point, a fraction of the XFEL pulse intensity is used at the timing tool branch to record its wavelengths spectrum as part of the timing information.

There have been some generally suitable schemes proposed.^26,28,29^ In this contribution, we present results on the basis of ultrafast melting of bismuth^30^ as an applicable technique for finding T_0_. Our approach is particularly applicable to protein fixed target experiments, which need a similar setup to be in place. Such a setup is schematically depicted in Fig. 1. Essentially, it needs an X-ray diffraction setup in transmission geometry. Additionally, an optical pump laser beamline needs to be in place serving as both a timing tool setup and a pump beam section with an adjustable beam path length.

## II. EXPERIMENTAL METHODS

Bismuth is a semi-metal rich in interesting properties resulting from a rather complex band structure. The energy gap between occupied states in the valence band (VB) and free states in the conduction band (CB) vanishes in certain crystal directions and is mostly so low that only small photon energies are needed to excite an electron to the CB. Hence, the absorption length for optical radiation is small and of the order of 7 × 10^5^ cm^−1^.^31^ Bismuth has 5 valence electrons per atom. Upon interaction with a short optical laser pulse, most of the absorbed optical power goes into the electronic system, which has a multitude of potential pathways for distributing the excess energy and temperature on various time scales.^32,33^ If the absorbed optical power does not surpass the energy per atom required for melting, reversible lattice dynamics ensue.^26,34–36^ However, if the absorbed optical power is sufficient and the lattice dynamics irreversible, two main pathways transfer the (poly-) crystalline ordered state to a disordered liquid state with higher entropy. This liquid state is reached by either the redistribution of sufficient thermal energy to the lattice via electron-phonon scattering or by the presence of excited electrons in anti-bonding states. The loss of order can be traced by observing the fading of the intensity in the Bragg peaks of coherently diffracted X-rays as a function of time. Complete fading, depending on experimental conditions, can be accomplished well within an interval of a picosecond^30^ and hence can be exploited to accurately determine T_0_. The timescale on which a Bragg peak fades is most generally dependent on the total amount of excited hot electrons. This is independent of whether the melting process is thermal,^37,38^ via an electron plasma quickly thermalizing with the lattice, or non-thermal by driving the lattice apart due to the excitation of non-bonding atomic states.^30,39–41^ Both processes individually, and eventually a combination of them, can result in an ultrafast disordering of the lattice. In the case of Bi, a definitive speed limit has not been found so far. This would manifest itself as a settling time constant for the fading even with an increasing fraction of excited electrons or laser pump fluence, as found in the semiconductor indium antimonide.^42^ One could argue a time limit is given by the half period of the A_1g_ lattice phonon mode in Bi.^30^ However, these initial studies of this phenomenon also showed that the nonthermal melting dynamics became linearly faster with higher fluence (within the limited accessible pump fluence range) with no saturation effect in the speed of the modified electron distribution in driving atomic motions.

**FIG. 2.**
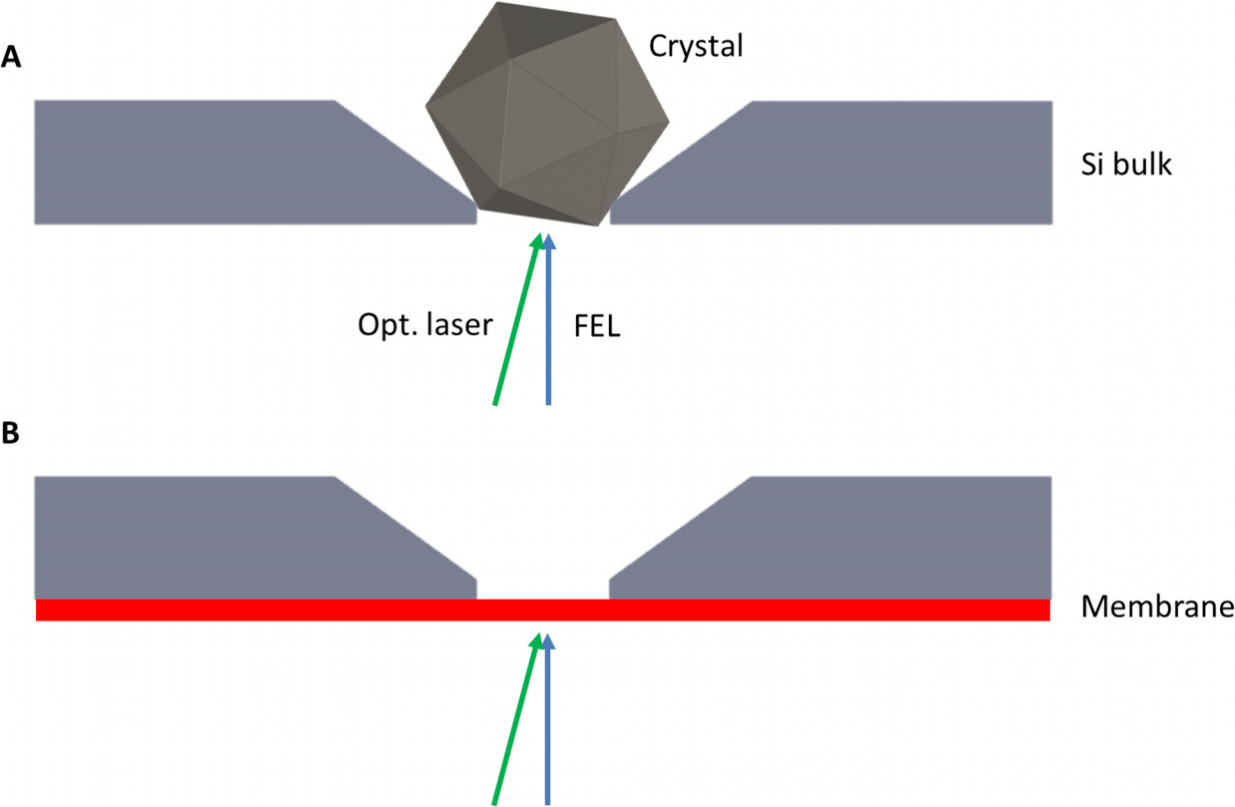
(a) To perform serial crystallography, chip features are designed to match the target protein crystal size. Dynamic flow when loading the chip results in random orientations for sampling sufficient reciprocal space in diffraction. The crystal rests in an etched pocket with openings to the up- and downstream direction of the FEL beam. (b) In the configuration to measure T_0_ a silicon oxide (or likewise silicon nitride) layer (thickness: 30 nm to 200 nm) remains on the upstream side of the chip by altering the manufacturing protocol. Leaving this membrane in place allows for coating with a thin layer of Bi (<80 nm), which can be investigated for its ultrafast melting dynamics. Dimensions are not to scale.

**FIG. 3.**
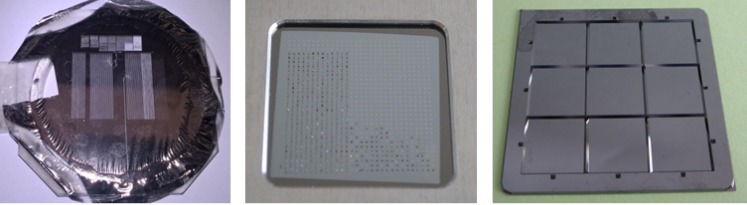
(Left) 50 nm Bi on 4 *μ*m Prolene substrate attached to a frame for handling. A column-like structure is clearly visible from a vast number of positions which have been probed by optical laser and FEL. (Center) A target with a 30 × 30 matrix of silicon nitride membranes of 30 nm thickness etched into silicon bulk. The left and bottom half of the windows have been partly shot leaving a destroyed membrane. (Right) Silicon oxide membranes of 200 nm etched into silicon bulk, with a total of 25 281 individual membranes arranged in 3 × 3 sub-matrices of 53 × 53 membranes.

The experiments have been performed at the SACLA free-electron laser,^43,44^ where we used the beamline of experimental hutch 2 (EH2) for our compact endstation.^45^ This setup has been developed to perform serial femtosecond crystallography (SFX).^46^ The majority of the conducted SFX experiments rely on a liquid jet injector^46–48^ to provide a continuous stream of embedded individual micro- to nanosized protein crystals to the interaction point. In our experimental scheme,^49,50^ the crystals are placed at particular positions pre-defined by nanofabrication of tailor made trapping sites into a silicon wafer; see Figs. 3(d)–3(e) in Ref. 49 and Fig. 2 of the present document. During data recording, all positions (summing up to about 20 000 on a single chip) of this crystallography chip (CC) are subsequently visited by the FEL and a single shot diffraction pattern is recorded at each position.

Figure 2 schematically depicts a single micron-sized protein crystal resting in one of the single wells as typically arranged in this type of fixed-target SFX. The size of the pocket is normally optimized to the crystal dimensions of the sample under investigation. This procedure usually involves a target crystal size using different crystallization conditions to give the best absorption at the excitation wavelength, highest fraction excited within constraints to keep the excitation fluence low enough to avoid peak power, multiphoton artifacts in the excited state preparation. The chip well features are then matched to the mean of the crystal size distribution to achieve this condition. For SFX experiments, the wells are open to both sides. One of the last steps in the production of the CC is the removal of a thin SiO_2_ membrane covering the side with the smaller opening. Leaving this membrane in place, provides a version of the CC suited to infer T_0_ between an optical pump laser and the FEL probe at the very same location of the protein crystal. For this, we coat the outer surface of the membrane with a thin film of polycrystalline bismuth and exploit the ultrafast melting of the lattice of that layer upon excitation with an optical laser.

**FIG. 4.**
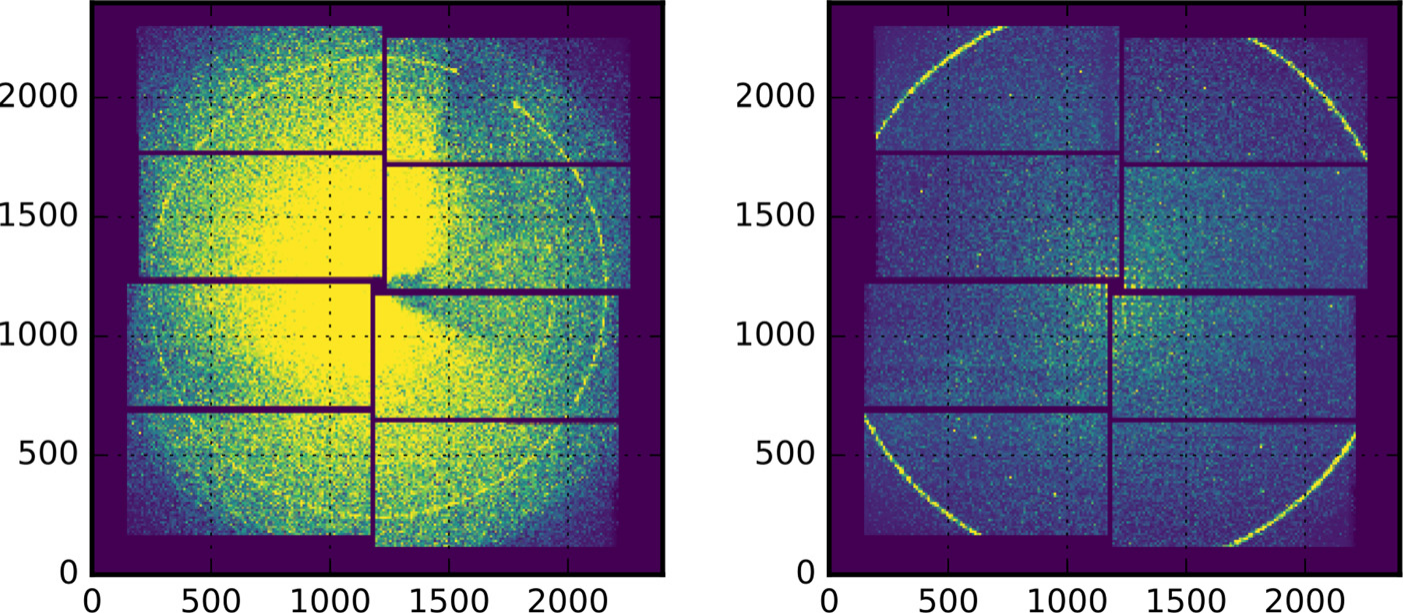
Single shot 2-D raw data from SACLA MPCCD-8 detector.^53^ (Left) 50 nm Bi sample on 4 *μ*m Prolene substrate. (Right) 50 nm Bi on 30 nm silicon nitride substrate. The most distinct diffraction ring is from the (002) reflection in Bi.^51^ Please note the enhanced background level of the Prolene substrate and that the detector distance is not the same for the two recordings.

**FIG. 5.**
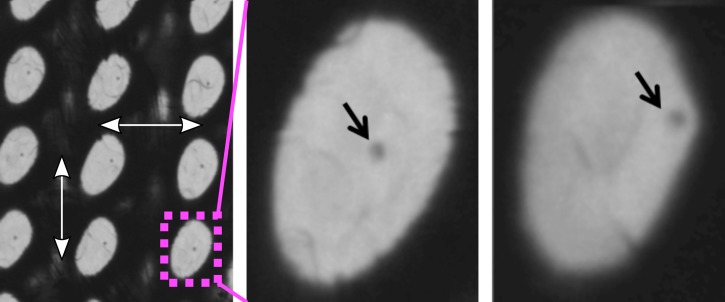
Light microscope images of a Bi target on a Prolene support. (Left) Sample section damaged by several optical laser shots. Dark area corresponds to intact Bi film, light area indicates damage inflicted in the Bi layer due to ablation by the optical laser. The lateral distance between subsequent shots is 200 *μ*m (white arrows). The optical laser has an elliptical beam shape. Imprinted is the size of the optical laser where the intensity is above the ablation threshold. (Center and right) Two zoomed in examples of single shot damage with good alignment of the FEL with respect to the optical laser and unfavorable alignment at the edge of the ablation area. The dark spot marked by a black arrow is the probing position of the FEL beam. The degree of alignment and hence the local laser intensity at the probing location is imprinted on the target on single shot basis during a T_0_ determination run.

In order to make the T_0_ determination approach applicable for a broader set of experimental situations, we also produced and measured other realizations of suitable bismuth targets. These targets should be much easier to prepare by the interested user. Such samples include commercially available silicon nitride membranes arranged in a matrix and coated by a thin bismuth film (see Fig. 3). A very cost-effective target can be built by coating a thin foil (4 *μ*m) of Prolene (a polypropylene structure), readily commercially available for widespread applications with X-rays. In case of the Prolene samples, the bismuth diffraction data show a lower signal-to-noise ratio (SNR) due to a significant Prolene background (see Fig. 4) when compared to data obtained from the micro-patterned chips. Nevertheless, its use can offer distinct advantages: First, it is a continuous target not restricted to prescribed positions and hence does not need highly advanced positioning. It can be produced in large sizes and amounts at very low costs allowing for a large total number of shots resulting in a statistical significance counterbalancing the reduced sample and diffraction quality. Second, the individual shots of optical laser and FEL do not fully destroy the shot area but preserve it in a condition where the FEL-optical laser spatial overlap and the damage inflicted can be investigated for each shot if needed, as shown in Fig. 5. By this, one can infer confirmation on the size, shape, and pointing parameters of the two photon beams. We find a (complete) ablation threshold of around 24.5 mJ/cm^2^ local laser intensity impinging on the target. Such information is furthermore useful to check if optical laser and FEL are in spatial overlap and, more specific, if this overlap is changing during a T_0_ run. If so, the data needs to be treated with caution since very different laser fluence conditions at the varying relative position of the FEL focus might affect the data interpretation. Moreover, the relative movement of the beams can result in timing errors due to path length changes. Last, it appears that the crystallites of Bi formed during the thermal evaporation coating process on top of the Prolene foil have somewhat reduced tendency to fully orient the [111] direction perpendicular to the surface. Such an orientation along [111] is an expectable characteristic of thin Bi layers deposited thermally onto a substrate. A direct consequence of the smaller degree of orientation is the appearance of otherwise suppressed Bragg peaks. That gives potentially access to a broader set of analyzable diffraction peaks enabling the time-resolved analysis of several peaks to infer T_0_.

**FIG. 6.**
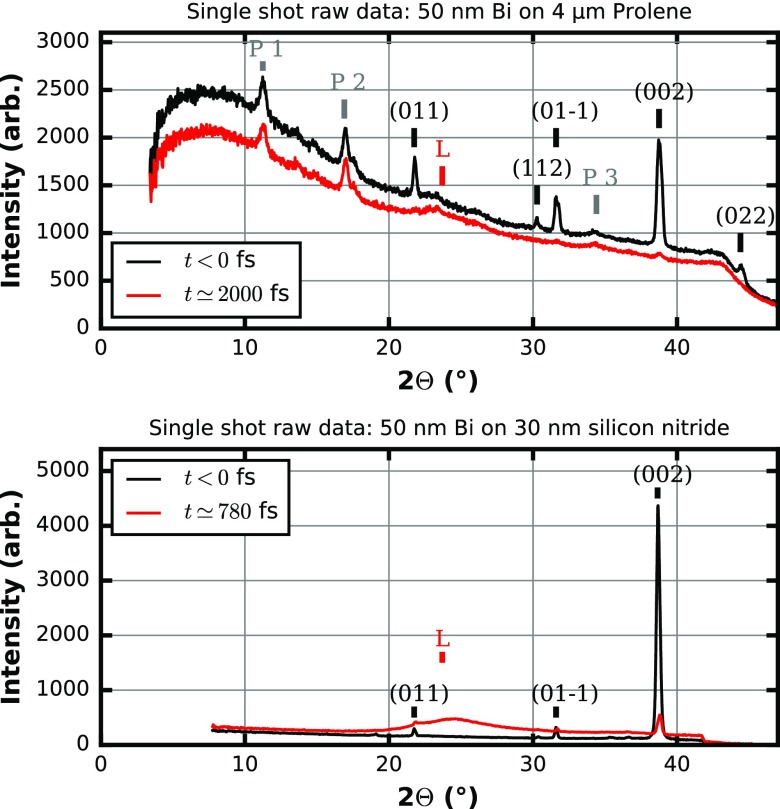
1-D representation of Bragg-angle Θ vs. intensity of single shot data by radial integration of the 2-D raw images for two different samples and two pump-probe time points, respectively. The scattering features are labeled by P if related to Prolene, by the corresponding Miller-indices according to Wyckoff *et al.*^51^ if related to the Bi lattice and with L for the signal of a liquid Bi phase. The visibility and intensity of the peaks are dependent on the X-ray energy and change at a different wavelength. (Top) 50 nm Bi sample on 4 *μ*m Prolene substrate: Two different time points for the pump-probe delay of 2000 fs after T_0_ (optical laser first) and negative time delay (FEL probe first, no dynamics). (Bottom) 50 nm Bi sample on 30 nm silicon nitride substrate: Two different time points for the pump-probe delay of 780 fs after T_0_ (optical laser first) and negative time delay (FEL probe first, no dynamics). The onset of melting can be readily observed in a single shot.

Figure 6 depicts a typical single shot signal for a selection of two different types of 50 nm thick Bi targets, namely, a continuous 4 *μ*m Prolene substrate (Fig. 3 left) and a 30 nm silicon nitride substrate (similar to Fig. 3 center). This single shot data is produced from radially integrating single shot two-dimensional (2-D) raw images of the diffraction intensity, as depicted in Fig. 4. Besides projecting the 2-D diffraction intensity distribution to a one-dimensional representation as a function of the scattering angle (twice the Bragg angle) only very limited data operations are performed. There is some masking of regions with unusual pixel values and of regions covered by a beam block used to prevent the optical laser beam hitting the detector. Independent of the sample details, the set of six reflections {00 ± 2} labeled according to Wyckoff *et al.*^51^ (using a rhombohedral unit cell) with a lattice spacing of d = 0.18684 nm is the best choice for analyzing the dynamics with respect to a T_0_ determination since this group of reflections offers sufficiently small lattice spacing. Typically, according to basic Debye-Waller theory, the influence on the intensity of diffraction peaks of a heated lattice becomes larger with smaller spacing d or larger scattering angles. Furthermore, the {002} reflections offer more favorable signal-to-noise properties via the matched Bragg condition at X-ray energies around 10 keV. Due to the alignment of the sample along the [111] direction, which is also the direction of the incident FEL pulse, the {002} planes align with an angle of about 18.35 degrees with respect to the FEL axis. This angle is close to the Bragg-angle of 19.38 degrees for the {002} planes. Due to misalignment of the bismuth crystallites with respect to the [111] direction, we find that the Bragg-angle at this wavelength of the FEL does not need to be perfectly matched to find large diffracting power for the {002} planes. However, if the FEL operates at a very different wavelength, the {002} reflections might be absent and other planes possibly dominate the diffraction pattern. For lower X-ray energies the {022} reflections will become more favorable reaching a matched Bragg-condition at energies around 6 keV. On the higher energy side, the {1–12} planes will provide a strong signal around 18 keV. Depending on the alignment of the crystallites in the sample, exploitable diffraction peaks should be readily available for the complete range of typical energies of X-ray FELs.

**FIG. 7.**
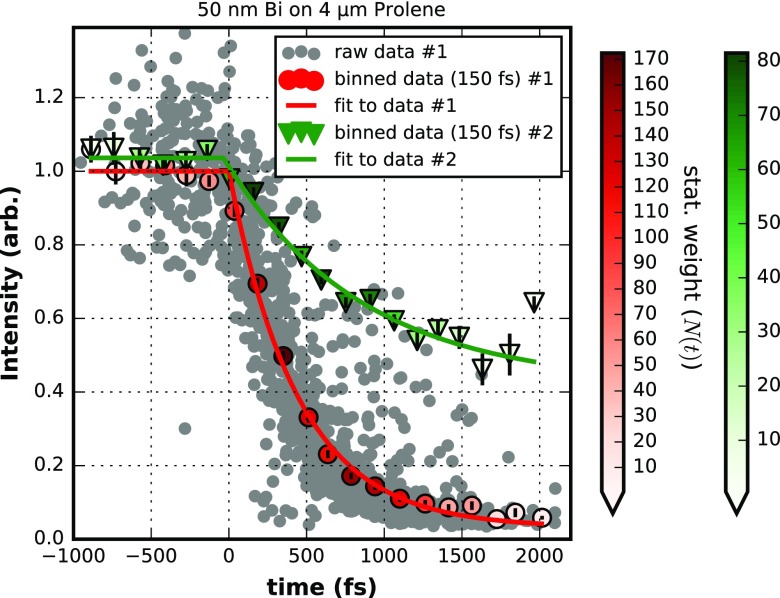
Dynamic data of the (200) peak diffraction intensity from 50 nm Bi on 4 *μ*m Prolene support for two different laser fluences of 45 mJ/cm^2^ (data #1) and 30 mJ/cm^2^ (data #2). For the dataset #1 the raw data is depicted for reference. Binning in 150 fs intervals produces data which is fitted by a piecewise exponential decay (see text). Fit parameters of dataset #1: tau = (443 ± 27) fs, T_0_ = (0 ± 12) fs, a = 1.0 ± 0.014, c = 0.03 ± 0.017; dataset #2: tau = (880 ± 170) fs, T_0_ = (43 ± 34) fs, a = 1.038 ± 0.013, c = 271 ± 38. The color coding of the binned data points represents the statistical weight, which is the number of single shots summed in a bin. Error bars attached to the binned data are deduced from the standard errors of the mean and for reference only. They are not used during evaluation. The Y-error of the single raw data points is around ±0.2. The X-axis is arbitrary in offset and here calibrated by dataset #1.

**FIG. 8.**
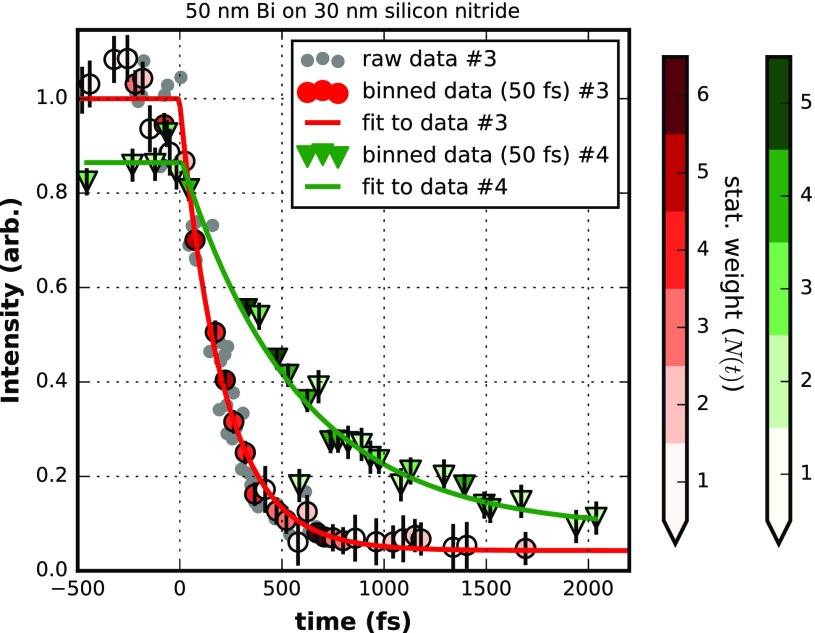
Dynamic data of the (200) peak diffraction intensity from 50 nm Bi on 30 nm silicon nitride support for two different laser fluences of 150 mJ/cm^2^ (data #3) and 60 mJ/cm^2^ (data #4). For the dataset #3 the raw data is depicted for reference. Binning in 50 fs intervals produces data which is fitted by a piecewise exponential decay (see text). Fit parameters of dataset #3: tau = (213 ± 12) fs, T_0_ = (0 ± 8) fs, a = 1.000 ± 0.012, c = 0.043 ± 0.010; dataset #4: tau = (571 ± 67) fs, T_0_ = (14 ± 32) fs, a = 0.865 ± 0.021, c = 0.087 ± 0.03. The color coding of the binned data points represents the statistical weight, which is the number of single shots summed in a bin. Error bars attached to the binned data are estimates from the fit results and for reference only. They are not used during evaluation. The Y-error of the single raw data points is around ±0.05. The X-axis is arbitrary in offset and here calibrated by dataset #3.

We recorded experimental data with an XFEL wavelength around 0.124 nm (10 keV) and an average shot energy of about 0.4 mJ. The FEL was horizontally polarized while the optical laser, the output of an optical parametric amplifier (OPA), was circular polarized and operated at 537 nm with a pulse length between 70 fs and 100 fs full width at half maximum (estimated according to specifications, typical experimental results are around 70 fs fwhm). The choice of wavelengths and polarization were constrained by the requirement of a pump beam in the green to trigger dynamics in a protein that was the primary objective of the beam time allocation used to master this approach of finding T_0_. Figures 7 and 8 depict results obtained from a 50 nm thick Bi film on a 4 *μ*m Prolene support and on a 30 nm silicon nitride support for a total of four individual T_0_ runs. The structural dynamics of the intense (002) diffraction peak has been analyzed for these runs. The two individual runs for each sample have been conducted with different optical pump laser fluences to allow observation of the effect of different levels of excitation. The time constant governing the melting of the sample is dependent on the fraction of excited carriers of the valence band into the conduction band upon absorption of the pumping pulse and hence the pumping laser fluence. The determination of the optical photons absorbed in the sample are given by the laser fluence impinging on the sample taking into account the reflected and transmitted contribution to the excitation interaction with the sample. The reflected relative intensity can be as high as 53% and is strongly affected by details of the particular sample.^31,52^ The fully transmitted intensity is typically at the percent level for a 50 nm Bi sample. If each absorbed photon of 537 nm wavelength would excite one of the five valence electrons to the CB, volume averaged excitation levels in the range of 30%–45% of the valence electrons would be reached with the highest fluence of 150 mJ/cm^2^ used for some of the data presented. Although crucial for the understanding of the melting process itself, the particular excitation levels are of a minor concern for the application as a T_0_ tool.

## III. DATA ANALYSIS

The data in Figs. 7 and 8 share the following characteristics: a faster (red markers) and slower (green markers) fading of the recorded intensity of the (002) diffraction is depicted. For the sake of clarity, only for the faster decay (red markers) the single shot raw data (grey dots) is plotted. A single data point (grey dot) is obtained by integrating the background-corrected intensity around the (002) reflection (see Fig. 6) of a single shot 1-D representation. The timing information of each single shot is taken from the timing tool^8^ (see Fig. 1). This timing is just a relative timing between the optical laser source and the FEL at the position of the timing tool. However, this information allows sorting the single shots with respect to delay times (e.g., small to large). By design, the order of the delay times as well as time differences between two delay times are preserved with respect to the timing at the location of the interaction point of the experiment, although the true delay times are unknown in value. With the evaluation of a T_0_ run, physically meaningful delay times at the interaction point will be finally determined.

The data represented by red or green markers are created by binning (bin width 50 or 150 fs) the raw data of single shots and are fitted (solid lines) with a piecewise exponential decay (PED) of the form

$$\begin{array}{ll} I\left(t\right)=a+c & t<T_{0}\\ {I\left(t\right)=a\;*\;e}^{-1/\tau (t-T_{0})\;}+c & t\geq T_{0}. \end{array}$$

We note that the transition region at T_0_, from the non-dynamic plateau into the actual dynamic decrease in intensity can be expected to be more complicated to describe in detail^42^ and the simple PED fit could fail for certain levels of accuracy, in particular, at higher laser fluences. The details will depend on the pulse length of the optical pump laser and possibly on the pulse length of the probe pulse if it is of considerable length; with around 10 fs FWHM, the FEL pulse at SACLA is so short that there is no need to de-convolute the data. Most notably, the details around T_0_ are governed by the dynamics of the atomic bond softening with the increased fraction of electrons in an excited state within the course of the optical pump pulse. This is something which needs to be investigated more closely in future experiments.

The data presented in Fig. 7 show that a reasonable determination of T_0_ is possible with laser photon fluences as low as 30 mJ/cm^2^. Note that the reported fluences are the impinging fluences at the probing position assuming a 2-D Gaussian distribution for the pump laser (see Fig. 5). Since the probing area is much smaller than the optical laser spot size the excitation is homogenous in lateral direction. A fluence of 30 mJ/cm^2^ impinging on a 50 nm thick Bi layer coated on a 4 *μ*m Prolene substrate does not fully melt the complete layer of Bi, but only a considerable fraction of it as the intensity of the Bragg peak settles substantially above zero. With such fluences, the fading of the Bragg peak intensities takes a comparably long time—here the lifetime is around 880 fs. At 40–50 mJ/cm^2^ laser fluence impinging on the target (with about 30% reflection losses) shown for the dataset number #1 of Fig. 7, the decay is already much faster (443 fs) and the Bragg-peak intensity fades out to zero indicating melting and basically an ablation of the whole Bi layer. The observed dynamics place the strongly driven phase transition in the nonthermal melting regime but even lower fluences bordering a fully thermal process are sufficiently fast enough with large changes in signal intensity to provide T_0_ determination in the 10 fs range, or sub-100 fs. Figure 7 shows that the determination of T_0_ by a PED is almost unaffected by the intensity of the pump laser. This is reflected by the fact that dataset numbers #1 and #2 give T_0_ values, which differ only within the statistical uncertainties of the two fits that is T_0_(#1) = (0 ± 12) fs and T_0_(#2) = (−43 ± 34) fs. The T_0_ value for dataset #2 is given relative to the T_0_ value inferred from dataset #1, which has been defined to be zero femtoseconds without loss of generality.

Dataset #1 of Fig. 7 originally consists of 1515 single shots of which only 1488 were selected and depicted since they survived discrimination against obvious quality constraints such as FEL intensity or optical laser power. In principle, a collection of 1515 shots for a determination of T_0_ takes less than a minute if the FEL has a repetition rate of 30 Hz or higher and the positioning system moving to a fresh spot between the shots can cope with this speed. However, it is very likely that some parameters, in particular the pump-probe delay, need to be changed during the collection of the shots to record data which spans the relevant regions of the time axis. In our case, the 1515 data points have been collected with six different nominal time delays between −500 fs and 2000 fs. Due to the 350–450 fs root-mean-square (rms) jitter per set delay, the whole time range from −500 fs to 2000 fs is filled with data. The total data recording time required was below 10 min (450 s) including adjustments and changes. This is a reasonably short time to measure or confirm such a crucial parameter like T_0_ for any time resolved experiment.

The data of Fig. 7 have been binned to 150 fs intervals. Averaging with respect to the x and y coordinates of the single shot data within the bins leads to the coordinates of the combined data (only bins with more than one single shot are kept). The number of single shots in a bin is used as relative weight within the least-squares fitting procedure by the common Levenberg-Marquardt algorithm. Alternatively, without significant influence on the results, the standard error of the mean intensity of a bin can be used as its error bar. As expected for sufficient SNR in the diffraction data and a well-chosen model function, the fit to the binned data and a fit to the non-binned data differ on the noise level of (7 ± 7) fs. From visual inspection the raw data seems to have a considerable variation. In some regions, e.g., between 500 fs and 1500 fs, this particular dataset shows some outliers towards more positive intensity values. These outliers survived initial discrimination versus monitored parameters of the experiment and seem to be beyond a statistical error of about ±0.2 for the single shot diffraction intensity. We believe that the origin of such outliers is most likely sample related, e.g., sample flatness, and there are probably technical measures in sample mounting to significantly remove them in the future. However, removing outliers by using various sigma cutoffs did not result in changes of the fit results. Therefore, the binned version shows that the data behaves statistically well and a dataset of the order of 1500 single shots is sufficient to exclude statistical sources of error as a significant contribution to the accuracy of the procedure.

The data of Fig. 8 are taken for 2 laser fluences and a 50 nm thick Bi sample on a 30 nm silicon nitride support. Both datasets consist of about 110 individual shots. Due to the better signal to noise of those targets, significantly fewer shots are needed to acquire a full decay curve of the Bragg peak intensity. Again, as in the case of the continuous Bi on Prolene (BoP) samples, two T_0_ measurements taken about 30 min after one another and taken for different effective decay times at different fluences give matching T_0_ results. The two values differ by less than 15 fs and hence well within the error bars of the individual fits to the data, which have a standard deviation of 32 fs and 8 fs, respectively. At a fluence of about 60 mJ/cm^2^, we find a 1/e decay time of 571 fs and at a fluence of 150 mJ/cm^2^ a time of 213 fs. Certainly, if possible, a higher fluence is beneficial for the determination of T_0_. The experiments are performed in a single shot fashion, and there exists no upper limit for the fluence as the local disintegration is anyhow inherent to the experimental procedure. The key is that the sample costs are really minimal and large surface area samples can be used to repeatedly perform T_0_ confirmation during the course of an experiment. Given the large changes in diffraction intensity at even modest fluences, the time to perform the T_0_ measurement with proper automation could be on the order of 1 min, making this a very attractive T_0_ tool for most experiments.

We note that the observed dynamics of the Bragg peak intensity could possibly be affected by forces from light pressure inhomogeneity^33^ due to the exponential decay in the optical absorption depth from the surface with inhomogeneous contributions to the melting process. For the scope of this experiment, it does not particularly matter what influences the fading of the diffraction peak intensities, but whether it can be exploited for an accurate determination of T_0_. The change in diffraction signal must be fast enough, reproducible, and produce a large enough change in intensity to give sufficient SNR for as short sampling times as possible to obtain T_0_ within the desired accuracy. These conditions hold for the Bi timing tool as demonstrated in the data presented. Systematic errors which could be based on an incomplete understanding or description of the true physics of the melting process, in particular, around T_0_, are of course difficult to quantify. But since the statistical behavior is quite robust in the timing data, the accuracy is sufficient for the present purposes. The T_0_ position accuracy can be further improved and corrected anytime a refined understanding is at hand. Presently, we would estimate our timing being absolutely accurate down to 50 fs, which is half the FWHM pulse length of the pump laser. In a more conservative approach, a timing accuracy estimation to maximally 150 fs appears reasonable. That would be up to 3 times our bin-width. The T_0_ determination is of course dependent on the timing information of the employed timing tool. Only certain systematic contributions to the timing tool accuracy could potentially find their way into the error budget of the T_0_ determination, using this feature for jitter correction. Given a reported rms precision of below 10 fs (Ref. 8) for the employed timing tool, the influence seems very small.^29^

While the micro-structured samples typically show a narrower statistical distribution around the mean values and by such require fewer amounts of total shots for a determination of T_0_, their production requires special resources and can be financially expensive ($200–300 per piece) if large amounts are needed. Furthermore, the alignment of such non-continuous samples is more challenging and hence potentially more time consuming to setup for a T_0_ determination run. The continuous BoP samples, on the other hand, are easy to fabricate if facilities for thermal coating are accessible. The target is continuous, and the alignment procedure is very quickly performable if a set of xyz translation stages are in place. We presently produce Prolene targets with a usable area of (30 × 30) mm^2^ which allows for 4500 shots if a 200 *μ*m pitch distance between the shot locations is used. This pitch is of course related to the size of the optical pump laser plus some safety margin.

Out of technical necessity, the experiments have been performed with an angle of roughly 10 degrees between the optical laser and the FEL axis (see Fig. 1). In such an arrangement, any misalignment of the travel axis of the X-Y-Z stages could translate to a change of the laser-FEL-overlap as a function of sample position. Hence, the alignment needs to be checked repeatedly over the course of a T_0_ determination. This is particular easy by investigating the imprints as illustrated in Fig. 5 for the BoP sample used. It is relatively easy to observe shifts of the FEL position relative to the optical laser such that this tool can also be used for characterizing beam pointing instabilities as well as higher accuracy correction for timing.

It needs to be tested, particularly for the BoP samples, if a scheme utilizing an additional FEL single shot prior to the pump-probe sequence can be used for an improved normalization. A major contribution to the statistical variance stems from a somewhat less homogeneous sample quality, which is much more a consequence of the Prolene support surface microstructure than it is the coating process itself. The sample could be probed with an additional FEL pulse providing an individual normalization to each shot. Although in such a scheme only half of the FEL shots would create timing information, there could be an overall performance gain.

## IV. SUMMARY

In conclusion, we present a useful method to determine the temporal overlap between an optical laser and an X-ray FEL beam to find T_0_ and absolutely calibrate a timing-tool setup. The method is particularly interesting where setups in transmission Bragg geometry are already employed for a dynamical investigation such as in fixed-target serial femtosecond crystallography. Exploiting the irreversible ultrafast melting of Bi is a comparatively quickly applicable technique providing an accurate yet easy interpretable result due to a simple fitting function. Furthermore, the technique is applicable for a wide range of pump laser fluences as well as wavelengths. In this contribution we show its feasibility for green pump wavelength. From Ref. 30 it is known that the use of near infrared radiation around 800 nm wavelength is equally sufficient to drive the melting process. There is no indication of a fundamental problem if excitation wavelengths are further extended to the blue spectral range or even shorter wavelengths. From our lowest fluence data at 30 mJ/cm^2^, which seem to be sufficient for melting, we conclude that a T_0_ determination similar to our boundary conditions requires at least 8 × 10^16^ photons per cm^2^ absorbed by the Bi target (50 nm thickness) over the beam size of the FEL. With longer pump laser wavelength, the reflectivity of Bi increases while the absorption decreases. A reversed behavior is present if the wavelength is changed from green to shorter wavelengths. This needs to be considered in order to provide sufficient flux at different wavelengths.

It is a particular advantage if the determination of T_0_ can be performed under conditions close or identical to the conditions of the actual experiment requiring the calibration of the time axis, in particular with respect to the employed pump laser wavelength. In contrast to most approaches utilizing reflectivity or transmissivity changes in semiconductors, our method utilizes the more common order of typical XFEL experiments with the FEL being the probe and not the pump pulse.
